# Comparison of Home Use Tests with Differing Time and Order Controls

**DOI:** 10.3390/foods10061275

**Published:** 2021-06-03

**Authors:** Nahyung Lee, Jeehyun Lee

**Affiliations:** Department of Food Science and Nutrition & Kimchi Research Institute, Pusan National University, Busan 46241, Korea; whk719@pusan.ac.kr

**Keywords:** consumer test, acceptability, home use test (HUT), context, real setting, consumer behavior, snacking

## Abstract

Consumer tests are classified in terms of the location of testing as laboratory tests or central location tests (CLTs) and home use tests (HUTs). CLT is generally used in sensory tests due to the ease of test control, whereas HUT has higher validity because of real consumption. However, the lack of test control in HUT is a major issue. In order to investigate the error occurrence and efforts required to minimize errors, three groups of tests were designed differing time and order control and evaluation was conducted using six snacks with texture differences. Errors related to time, order, and consumer or sample number were higher for more controlled conditions, however, most errors were recoverable using identification information except for cases of no response. Additionally, consumers preferred to consume all snacks in the evening at home, which differed from the typical 9 a.m. to 6 p.m. evaluation time in CLT. However, the timing differed for consumers with self-reported snacking time. The research title that included the term ‘home’ might have influenced the participants’ choice of location for evaluation. Overall, there was no significant difference between the results of groups despite different time and order controls, which could increase the applicability of HUT.

## 1. Introduction

Acceptance tests are crucial for food companies as acceptability can be used as an estimation for the possible repurchase of products by customers. These tests are usually conducted at sensory laboratories under a controlled environment using samples and preparations, which have been used to predict potential long-term purchases [1]. Consumer acceptance tests are largely divided into laboratory tests or central location tests (CLT) and home use tests (HUT). In CLT, consumers visit a specific place such as a shopping mall, hospital, or sensory test lab for undergoing the test. Most of the external factors, except certain environmental attributes under investigation, can be controlled at these places. Thus, environmental control is important in sensory tests. Even though CLT is widely used, it is unreasonable to evaluate the whole product experience with a small serving presented in a relatively short exposure time. Thus, its validity has been questioned in the real world [2,3] because consumers are practically affected by a variety of environmental factors in day-to-day life. Hence, control elements of sensory tests have been improved and tested to reflect real-use environments for years [4,5,6,7]. On the contrary, HUT is conducted at home where the consumers can evaluate in natural circumstances, thus it is one of the most noticeable methods to measure acceptability in real consumption. Nevertheless, the biggest challenge of HUT is control, as the evaluation is autonomous and is influenced by external factors such as evaluation of an uncertain amount of sample, improper focus, and interference of other family members’ during evaluation.

In this respect, several comparative studies between CLT and HUT have been done as the context effect suggests that the results would be different depending on the test location [4,5,8,9,10]. Most studies drew higher scores from HUT as the consumers could evaluate naturally in a relaxed setting for a prolonged period [5,9,10]. On the other hand, participants in controlled settings approached the tests analytically, in order to detect differences among the provided samples in a better way [5,11]. In addition, Boutrolle et al. [5] suggested that the contextual effect was influenced by different types of products. Hence, using CLT would be a better option for the evaluation of samples with small differences and no relation to consumption. Even though HUT would be more relevant for the evaluation of sample types, as they would be more specifically related in certain contexts of consumption, there is not enough information on the implementation of HUT and their influence on data acquisition.

Several studies using HUT have dealt with the real situation in many ways, however, minimal information is available about the mode of implementation. Therefore, the information required to build structural standardization is inadequate. Accordingly, it is important to examine the various factors in HUT. The use of a maximum of three samples is encouraged for two reasons: better understanding of the experiment for participants and digression from the natural situation [12,13]. Sample sizes in HUT are larger than those of CLT as the evaluation period is longer. The samples provided for CLT are insufficient compared to natural consumption situations [5,12,13]. Some studies asked the participants to consume a minimum quantity of samples [5,9], however, most studies did not mention if they used any instruction regarding the quantity of sample consumed. Zandstra et al. [1] conducted a study for comparing consumption. The results suggested that the group of participants that received identical products continuously consumed lower amounts of samples than the free-choice group.

Product information on the package should be considered in HUT design. Consumers noticed package cues during repeated consumption [14]. Mahieu et al. [15] researched if the participants got the sensory description from wine labels. Conversely, using a container labeled with a three-digit code enabled not providing the original package of the sample to consumers [10]. However, perishable food required careful consideration regarding variables such as temperature [11] and repackaging may raise food safety concerns.

For procedures, samples were provided through the lab stopping by [1], visits to participant homes in order to provide next testing samples and collect their answered questionnaire [5], or using a delivery service [16]. Unfortunately, most studies just stated the delivery status as ‘delivered’ or ‘sent’, hence, handling of the samples was unclear. Participants in previous studies were asked to record their answers on a score sheet [16], however, currently, online questionnaires are being implemented [14,15,17]. In addition, photographs of the participants [10], videos with observations [18], or interviews [19] are also taken for evaluation. The sample order was randomly allocated. Zandstra et al. [1] compared acceptance depending on the degree of freedom to choose samples. Participants were asked to evaluate alone [1] or with family, friends, or both [17]. Zandstra et al. [1] allowed products with different ingredients to be used.

**Figure 1:**
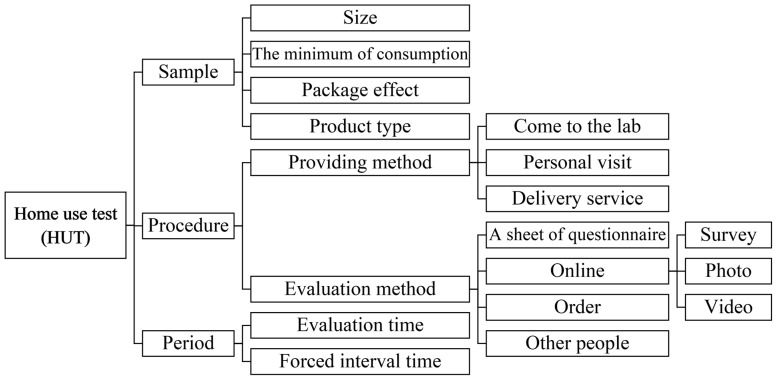
Summary of external factors in home use test (HUT).

CLT has a fixed time limit for participants to evaluate samples, thus, hedonic scores could be affected [5]. In contrary, HUT is conducted over a period of one week at least in order to help the participants develop an overall liking for the sample products by long exposure [13,20]. Thus, boredom [16] and familiarity with unusual flavors [21] were investigated. One of the greatest benefits of HUT is that the samples can be evaluated at any time, although it could be tested at certain time period [14]. Furthermore, after evaluating a sample, a forced minimum time lag before testing the next sample was set in HUT evaluations [10,15]. Some factors of HUT could be controlled to meet the aims of the test. A summary of external factors at HUT in the aforementioned paragraphs can be found in Figure 1.

Some challenges from the past remain, however, with the development of internet technology, the data can be collected and checked on a real-time basis. Furthermore, HUT is being used more owing to the prohibition of public gatherings due to the COVID-19 pandemic. These can be an alternative method of testing in the new normal era. Although sensory evaluation has started moving out of the controlled laboratory environment in order to reflect real consumption, COVID-19 accelerated the speed of change. HUT is a necessary form of consumer test. There is a lack of studies done on these tests, hence, they need to be investigated further for development and standardization. In addition, many studies mention the importance and common limitations of HUT, however, the information on methods for overcoming these limitations is not enough. Therefore, it is imperative to review the kind of errors that could occur while conducting HUT and find the amount of control/effort required to minimize the error.

The objectives of this study were as follows: (1) to determine if home-use tests could be an alternative to central location tests or laboratory tests, (2) to study the kind of errors that could occur while conducting home use tests, and (3) to suggest critical control factors.

## 2. Materials and Methods

### 2.1. Participants

A total of 300 Korean participants (218 females and 82 males between 19 and 65 years) were recruited through the online bulletin board in Pusan National University or word-of-mouth and enrolled utilizing an online survey tool (Survey Monkey, Palo Alto, CA, USA). Participants were asked to use QR codes. The demographic information of the consumers, along with their snacking frequency and usual snacking time on a daily basis, are shown in Table 1. Participants were selected based on the frequency of snack consumption (at least once every other day), willingness to consume samples, and absence of any food allergies and pregnancy. People were asked to choose items that they were not willing to consume as a snack from a list. Those who were unwilling to consume any of the test samples were automatically excluded from the study. The addresses of the participants were collected for shipping samples for testing at home. After the experiment, participants who completed the evaluation received mobile gift cards as compensation. This study was approved by the Institutional Review Board at Pusan National University (PNU IRB/2020_59_HR).

### 2.2. Samples

Jeltema et al. [22] divided individuals into four major groups depending on their mouth behavior: Crunchers, Chewers, Suckers, and Smooshers. Six commercial snacks (Table 2) with a clear difference in texture were selected according to mouth behavior. Samples were individually wrapped in a Kraft bag, labeled with three-digit random codes and consumer numbers. Participants received all samples and instructions at once by postal delivery.

### 2.3. Test Design

**Figure 2:**
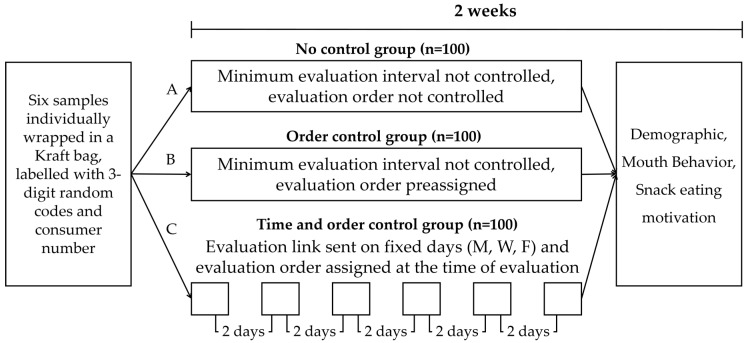
Schematic diagram of test design for comparing three home use tests with differing test controls.

Participants were divided into three groups. Each group had 100 participants and their sex, age, and address were considered in order to balance their proportion. If the address provided for delivery of samples was the same, we considered the participants to be living together and assigned them to the same group to minimize confusion during evaluation. Group A was the ‘No control’ group for time and order, thus consumers were allowed to proceed with the evaluation at any time and in any order they wanted. Group B was the ‘Order control’ group that consisted of participants who were instructed to evaluate in a preassigned testing order. However, they could evaluate whenever they wanted. Group C was the ‘Time and order control’ group that had a preassigned order and evaluated one sample every 2 days, three times per week. No instructions were given regarding the minimum amount of consumption and presence of other people with the participants while conducting the test. The instruction manual used pictograms in the description for ease of understanding. Participants were simply referred to quick response (QR) codes on the instruction manual or click links that were sent via text messages. The ‘No control’ and ‘Order control’ groups used QR codes provided on the instruction manual, whereas the ‘Time and order control’ group received messages including the link to the online questionnaire at 10 am on Monday, Wednesday, and Friday for two consecutive weeks. If the participant did not respond to the questionnaire for more than 72 h since the last evaluation, a reminder text message was sent to finish the assigned evaluation. Participants in the ‘Time and order control’ group were reminded 2 days later to maintain the evaluation intervals with other participants in the same group. The participant evaluation was terminated if they ignored the message three times consecutively. A schematic diagram of test design for comparing three home use test settings with differing test controls regarding evaluation time and test order is found in Figure 2.

#### 2.3.1. Questionnaire

Consumers filled out their identification number and product number each time and selected the time and location of their evaluation. The questionnaire was composed of queries regarding the product acceptability, texture characteristics, and prior knowledge of product. Overall acceptability, liking for the packaging, flavor, and texture were evaluated by using the nine-point hedonic scale (1 = “dislike extremely” and 9 = “like extremely”). Flavor intensity, texture intensity, and afterfeel intensity were measured using the nine-point scale (1 = “extremely weak” and 9 = “extremely strong”), whereas the amount of residue was measured on the six-point scale (0 = “None” and 5 = “very much”). Participants checked suitable texture terms using check-all-that-apply (CATA) and the mouth behaviors (cruncher, chewer, sucker, or smoosher) that were relevant to the corresponding sample were determined. Subsequently, they answered yes/no questions about knowledge of samples, brand name, and experience. Additionally, willingness to purchase was measured by using the five-point scale (1 = “I would definitely not buy” and 5 = “I would definitely buy”).

#### 2.3.2. Supplementary Questionnaire

After all the evaluations were done, participants were questioned about demographics, motivation of snacking, and mouth behavior. In snacking questionnaire, participants were questioned about three main parts. First was frequency, time, and reason for snacking. The second was a liking towards 11 snacks (snack, cookie or cracker, bread, fruit, chocolate, coffee, ice cream, beverage, jelly, nuts, and rice cake) using the nine-point hedonic scale (1 = “dislike extremely” and 9 = “like extremely”). Lastly, they were asked to respond to questions about the motivation of snacking using the six-point scale (1 = “never” and 6 = “always”) [23].

In the mouth behavior questionnaire, the participants were questioned for preference of mouth behavior. They responded to the degree of preference with the six-point scale (1 = “strongly disagree” and 6 = “strongly agree”) using questions that revealed the difference in the texture of food items, and photographs representing each of the four mouth behaviors [24,25,26,27]. They also responded to questions about the condition of their teeth and were asked to allocate the importance of taste, flavor, and texture by percentage.

### 2.4. Data Analysis

Data were divided into two categories: completed without error and error occurred. Their frequency was recorded. Data that did not have any errors was considered complete data. Data having errors related to time delay, evaluation order, entry of wrong sample number, or consumer identification were recovered by tracking personal identification information (last four digits of the phone number). Missing response cases were not followed up due to time passing after consumption. After confirmation, all data, except that of participants who did not respond, were corrected and used for analysis. Demographic information was completed with further requests from participants. Therefore, the number of participants whose data was available was different depending on the samples. The number of days taken for completion of evaluation with all samples was presented as mean, minimum, median, and maximum values of the difference between the start and end date using a data unit. The frequency and percentage for evaluation time and place, knowledge of samples, mouth behavior (MB), and amount of consumption were calculated. Liking and perceived intensity scores, willingness to purchase, and adequate portion size were analyzed using analysis of variance (ANOVA) to determine significant differences among groups and samples within each group. When significance was found, the Fisher’s least significant difference (LSD) was conducted as a post-hoc test at a significance level of 0.05. Additionally, the evaluated order from the ‘No control’ group was counted as the frequency.

Data from CATA was presented in terms of the frequency of selected sensory attributes and used for correspondence analysis (CA). The RV coefficient test was also performed using the results of CA to compare samples and terms between groups.

Statistical analysis was performed using SAS^®^ Software 9.4 (SAS Institute Inc., Cary, NC, USA). RV coefficient tests were analyzed using XLSTAT^®^ Software package (Version 2020.2.1 Addinsoft SARL, New York, NY, USA).

## 3. Results and Discussion

### 3.1. Checking of Errors and Analyzable Data

The frequency of analyzable data acquired from evaluations is shown in Table 3. Complete data indicates data collected correctly for the conditions of each group without any errors, including time delay. Overall completeness was the highest in the ‘No control’ group and was the lowest in the ‘Order control’ group. Completion of the survey was influenced by the degree of controls such as preassigned order and evaluation interval for each group. As the ‘No control’ group had the least control compared to the other two groups, only seven participants exceeded the evaluation period.

Simple errors such as the entry of incorrect consumer numbers and the three-digit random codes were detected and data was saved. In ‘Order control’ and ‘Time and order control’ groups, some evaluations were done without following test design protocols, however, their sample number was identified with data. When the evaluation was delayed, participants were reminded and the evaluation period was extended. In addition, no response was also counted as an error. The pattern of overall error occurrence was similar to that of completeness, with ‘Order control’ and ‘Time and order control’ groups having errors nearly twice as compared to ‘No control’ groups. ‘Order control’ group accounted for most errors from preassigned orders. However, a few also occurred in the ‘Time and order control’ group despite the notification. Additionally, the completeness from ‘Order control’ was the lowest, although the degree of control was not greater than that of the ‘Time and order control’ group as the preassigned order error was counted several times per person. When preassigned order error was treated as one per participant, despite its occurrence being more than once, then the order error occurrence was the highest in the ‘Time and order control’ group, followed by ‘Order control’ and ‘No control’ groups. Other errors were mostly related to incorrect entry of numbers, such as consumer numbers or three-digit random codes.

Recoverable errors referred to error data that was correctable. Even if the same consumer repeated mistakes more than twice, these were treated as an error. Participants had to enter the last four digits of their phone numbers for each evaluation, hence, their consumer numbers could be traced and errors could be rectified.

The sum of missing data in each group was in the following order: ‘No control’ group followed by ‘Order control’ and ‘Time and order control’ groups. For the ‘Order control’ group, missing data for apple sauce and potato chips was higher than the total number of dropped consumers. Moreover, participants who did not complete all evaluations were also in the order of ‘No control’, ‘Order control’, and ‘Time and order control’, however, some of their data were included.

Participants who did not complete the evaluation within the preset time period were considered for extended evaluation. Participants for extended evaluation from the ‘Time and order control’ group were considerably greater in number compared to the ‘No control’ and ‘Order control’ groups. For the ‘Time and order control’ group, the participants could not proceed to the next step on their own because they were informed of the testing sample within the evaluation intervals. Hence, the ‘Time and order control’ group had a lesser frequency in the no response and dropped consumers, although their number was highest in the extended evaluation.

All other errors were recoverable with confirmation, except for non-response data. The response rate for all groups was high and the frequency of error differed depending on the degree of control, which could be converted into complete data using the identification information.

### 3.2. Evaluation Time and Place

Information of evaluation time and place for each group is shown in Table 4. Most samples were evaluated in the evening except for jelly in the ‘No control’ group and candy in the ‘Time and order control’ group. Consumers in ‘No control’ and ‘Order control’ groups evaluated samples in the afternoon frequently. However, for the ‘Time and order control’ group, the frequency of evaluation was slightly higher in the morning, probably because the notification was sent in the morning. Samples in each group were evaluated with the least frequency at dawn. Depending on food types, it may have a more appropriate time of the day. Birch et al. [28] indicated that breakfast food items were preferred in the morning than in the afternoon, whereas food items associated with dinner were preferred in the afternoon than in the morning. CLT is normally conducted within a fixed time period, usually from 10 am to 6 pm, whereas participants of HUT choose appropriate consumption time according to their convenience unless noted otherwise, hence, natural behavior can be practiced. A comparison of the evaluation time for CLT and HUT shows that most HUT participants usually conducted the evaluation in the late afternoon or evening [9]. This led to increased satisfaction due to free conditions [5]. Comparison of liking categories depending on evaluation time showed no difference (*p* > 0.05). In this study, evaluation time did not influence acceptability.

All samples were mostly consumed at home, followed by the workplace. The snack consumption location for Canadians and Norwegians was home more frequently, followed by the workplace [29,30]. In addition, participants were informed about the ‘Home use test’ before the experiment. Most of them provided their home address for evaluation location as they might have thought that considering the name of the test, they had to evaluate at home. More than half of the participants were office workers, therefore, the evaluation location had a greater influence on the snacking time compared to the supplement questionnaire and real evaluation time.

### 3.3. Number of Days Taken for Home Use Test (HUT)

Table 5 shows the number of days taken for HUT with six samples by calculating the difference value between the start and end date. ‘No control’ and ‘Order control’ groups showed a similar pattern in the number of days taken including mean, minimum, and median values, whereas the ‘Time and order control’ group had a much higher value. When comparing the maximum number of days taken, ‘No control’ and ‘Time and order control’ groups had a higher value than the ‘Order control’ group.

The testing days were not designated, hence, ‘No control’ and ‘Order control’ participants could conduct the test in one day, whereas ‘Time and order control’ participants could take up to 10 days for completing the evaluation, considering the interval time and the date of sending a text message. Interestingly, some participants from the ‘Time and order control’ group communicated with their acquaintances in a different control group and received the survey link or QR code before their designated evaluation link was sent. However, we could not consider acquaintances in assigning participants into the same or different group. The instructions have to clearly stated that confidentiality should be maintained during and after participation, even if the participants are acquainted with each other.

The results of the maximum days taken by the ‘Time and order control’ group reflected the effect of the periodic testing intervals and reminders. One consumer from the ‘No control’ group took 26 days to complete the testing. The missing data were found a few days later and completed. The maximum number of days for completion of evaluation in the ‘No control’ group was 15 days without this data. However, several participants from the ‘Time and order control’ group who received the evaluation and supplementary questionnaire on the last day did not evaluate carefully. They completed only one of the questionnaires and the remainder was completed after the reminder. Another downside of sending reminders was that some participants from the ‘No control’ and ‘Order control’ groups completed all remaining evaluations at once after receiving the reminder because they thought that they were supposed to finish the questionnaires immediately. Furthermore, some participants lost the testing samples and requested to receive more samples. Therefore, they needed more time for testing.

### 3.4. Consumer’s Liking and Perceived Intensity of Samples

Table 6 presents mean values for consumer acceptability, perceived intensity, and amount of residue for participants of ‘No control’, ‘Order control’, and ‘Time and order control’ groups. The mean liking scores were generally between ‘Neither like nor dislike’ and ‘Like moderately’. In general, the liking, intensity, and amount of residue scores showed similarity among the three groups. Each liking category indicated very similar results for samples: spread wafer had the highest score in overall, package, and flavor liking category, while potato chips had the highest score in the texture liking category. Candy ranked the highest for after feel and texture intensity. Apple sauce scored the lowest in every liking category and intensity. The amount of residue showed the highest value for spread wafer and the lowest value for candy. There were no significant differences (*p* > 0.05) between groups in liking, perceived intensity, and amount of residue, while there were significant differences (*p* < 0.05) within groups.

Overall, liking was rated positively ranging between neutral to like moderately, probably because all products were commercially available [31] and the comfortable condition in context could have positively influenced acceptability [5,32]. Apple sauce is not available for sales in Korean markets, therefore, the Korean consumers were not familiar with the product. However, its taste and texture were liked as they are similar to other products, such as apple pie [33]. Considering the results, participants did not have much knowledge of apple sauce (Table 7). Additionally, many participants answered in open-ended questions that they would eat the remaining sample with other snacks such as bread or yogurt rather than eating apple sauce by itself.

Although spread wafer was also an unfamiliar product (Table 7), its liking score was relatively high, which could have been affected by brand awareness [34,35] and familiarity with the spread used for filling in the spread wafer. Soerensen et al. [36] indicated there was no dynamic liking when novel flavors were added because of a high perceived familiarity with chocolates. In addition, well-known samples were evaluated first, whereas unfamiliar samples were evaluated later in the ‘No control’ group (Table 7 and Table 8).

Although samples were wrapped in Kraft bags, consumers in the ‘No control’ group might have opened all bags to choose their evaluation order. More than half of the participants had knowledge of samples except for apple sauce. Although the spread wafer had low product awareness and experience, its brand awareness was high. On the other hand, apple sauce was mostly evaluated last and it had the lowest brand and product and brand awareness, and experience.

### 3.5. Purchase Intent and Price Willing to Pay

The results of purchase intent and price that the consumers were willing to pay are shown in Table 9. There was no significant difference between the groups (*p* > 0.05) and the samples showed significant difference within each group (*p* < 0.05). Similar to overall liking, spread wafer rated the highest in purchase intent, while apple sauce rated the lowest. Although apple sauce is similar to baby food, it was the only product not available in Korea, and thus was an unfamiliar product for the participants.

Participants were asked the price that they were willing to pay in Korean won (KRW) for a provided quantity of each sample as an open-ended question and the mean values (and SD) of the responses are shown in Table 9. There was a significant difference (*p* < 0.05) within groups while there was no significant difference (*p* > 0.05) between groups. All samples were rated similarly among the three groups, except potato chips and spread wafer in the ‘No control’ group. Participants were willing to pay the highest price for jelly and the lowest for candy. The problem was that candy, cereal bar, and spread wafer were provided in a quantity of more than one, thereby confusing the consumers whether the question was for only one piece or all provided. Most samples were rated higher than the original price except for potato chip and spread wafer (Table 2 and Table 9). For apple sauce and spread wafer, the difference of the values between the original price and the participants’ response was bigger than that of others due to participant’s unfamiliarity with the products.

### 3.6. Analysis of the Texture

#### 3.6.1. Mouth Behavior Used during Consumption

Consumers chose all relevant mouth behavior such as cruncher, chewer, sucker, or smoosher during consumption (Table 10). The highest frequency of mouth behavior for each sample was similar between groups. The mouth behavior commonly used for apple sauce and candy was sucker, and that for cereal bar, jelly, potato chips, and spread wafer was chewer, which was slightly different from the expected results (Table 2). Although the smoosher category included soft food, such as ripe bananas and custard, apple sauce was close to liquid, and a large portion of consumers answered that they could not feel any texture and drank it like a juice. Moreover, the chewer category was also selected because of tiny particles. Others had the highest frequency in the chewer category except for candy because its texture changed during eating. Jeltema et al. [22] mentioned that people perceived the overall texture of a food item as the texture that lasts the complete duration, rather than that at the beginning.

#### 3.6.2. Correspondence Analysis of Texture Characteristics

**Figure 3:**
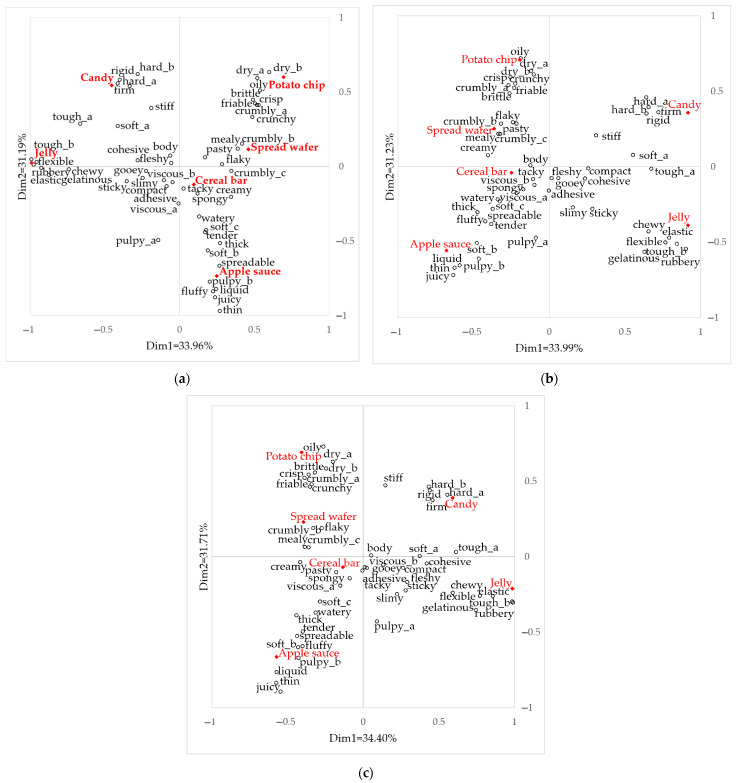
Correspondence analysis biplots using 51 texture attributes from the (**a**) ‘No control’, (**b**) ‘Order control’, and (**c**) ‘Time and order control’ groups. A total of 51 attributes were provided for CATA, Rhombus (◆) indicates samples.

**Figure 4:**
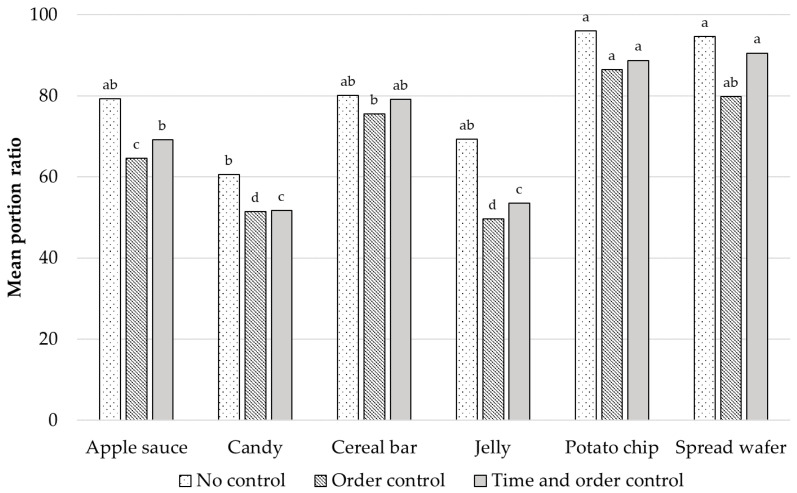
Adequate portion size evaluated by consumers. Adequate portion size was presented as an open-ended question (written down as the ratio value compared to the provided sample amount as 100). There are no significant differences between groups; samples sharing the same letter at the top of bars means no significant differences within each group (α = 0.05).

Correspondence analysis (CA) biplot shows the relationship between snack samples and the 51 texture attributes evaluated using the CATA method from each group (Figure 3). With Dimension 1 (Dim 1) and 2 (Dim 2), Figure 4a–c explains the 65.15% data variance in ‘No control’, 65.22% in ‘Order control’, and 66.11% in ‘Time and order control’ group. The RV coefficients provided that the terms configuration was similar between ‘No control’ and ‘Order control’ groups (RV = 0.963, *p* < 0.001), ‘No control’ and ‘Time and order control’ groups (RV = 0.961, *p* < 0.001), and ‘Order control’ and ‘Time and order control’ groups (RV = 0.955, *p* < 0.001). Samples configuration for the groups were as follows: ‘No control’ and ‘Order control’ group (RV = 0.989, *p* < 0.001), ‘No control’ and ‘Time and order control’ group (RV = 0.997, *p* < 0.001), and ‘Order control’ and ‘Time and order control’ group (RV = 0.984, *p* < 0.001). Samples with a difference in texture were dispersed into each quadrant and were explained by nearby texture characteristics.

### 3.7. Analysis of Portion Size by Consumers

The amount of consumption evaluated by consumers is shown in Table 11. All groups indicated similarity. More than half of the participants consumed all the provided quantities of cereal bar, potato chips, and spread wafer, whereas more than 40 participants consumed under 1/3 of the provided quantity for apple sauce, candy, and jelly. Liking was positively related to consumption [37,38]. However, in our study, overall liking was high for candy and jelly, while their consumption was low. This might be related to the time required to intake these food items because of their texture attributes (Figure 3). Furthermore, the adequate portion size evaluated by consumers was lower than the provided quantity when samples provided were considered as 100 percent (Figure 4). There was a significant difference (*p* < 0.05) within groups while there was no significant difference between groups (*p* > 0.05). The quantity of samples provided in CLT is relatively smaller than that of HUT along with a brief exposure time [39], hence, the prediction of the amount of consumption in a laboratory setting could be missed out. Gough et al. [40] found that participants might underestimate the portion size consumed in laboratory settings because of a tendency to conceal their eating behavior.

### 3.8. Suggestions for the Home Use Test and Limitations

Contrary to CLT, many external factors influence testing in a real setting; hence, greater efforts are required for the evaluation of several samples in HUT. After follow-up, the final number of participants with completed data was high, despite the occurrence of various errors. Most errors were related to incorrect entry of consumer numbers or three-digit random codes. Other errors included not following the preassigned order of testing or evaluation period extension. The consumer and sample numbers were both three-digit numbers. This might have confused the participants as they had to enter these numbers directly using open-ended questions, despite receiving six samples at once. Nevertheless, identification information helped in the modification of errors.

Kraft bags were used for hiding packages of samples before evaluation. However, it may not have served the purpose of random sample selection in the ‘No control’ group as familiar and/or preferred products were evaluated first. Moreover, a few participants unwrapped Kraft bags as soon as they received them, thereby mixing the sample numbers. In such cases, repackaging was required until the effect of packaging was studied and consistency of the food quality was ensured by avoiding external factors such as temperature, long contact with humid air, or direct sunlight. Moreover, it is important to check if the shelf life of the product would last an extended evaluation period.

Six samples were provided simultaneously by postal delivery before the start date. However, some participants from the ‘No control’ and ‘Order control’ groups conducted the test before the announcement of the start date using the QR code on the instruction manual. On the other hand, the ‘Time and order control’ group could start testing only on the day the survey link was provided. The survey link should be provided for the first sample evaluation if the start date of the evaluation is to be fixed as it eases the follow-up process.

In this study, new findings were that the job profile of the participants and the time of sending messages could influence evaluation time. The term ‘home’ used in the research title in the testing information may have influenced the evaluation place. Accordingly, the QR code allowed more freedom than the link in terms of evaluation time. In addition, the access time in online evaluation did not coincide with evaluation time in the questionnaire despite its mention in the written instructions. Hence, it was important to emphasize the instruction prominently or by using a video for better understanding.

**Figure 5:**
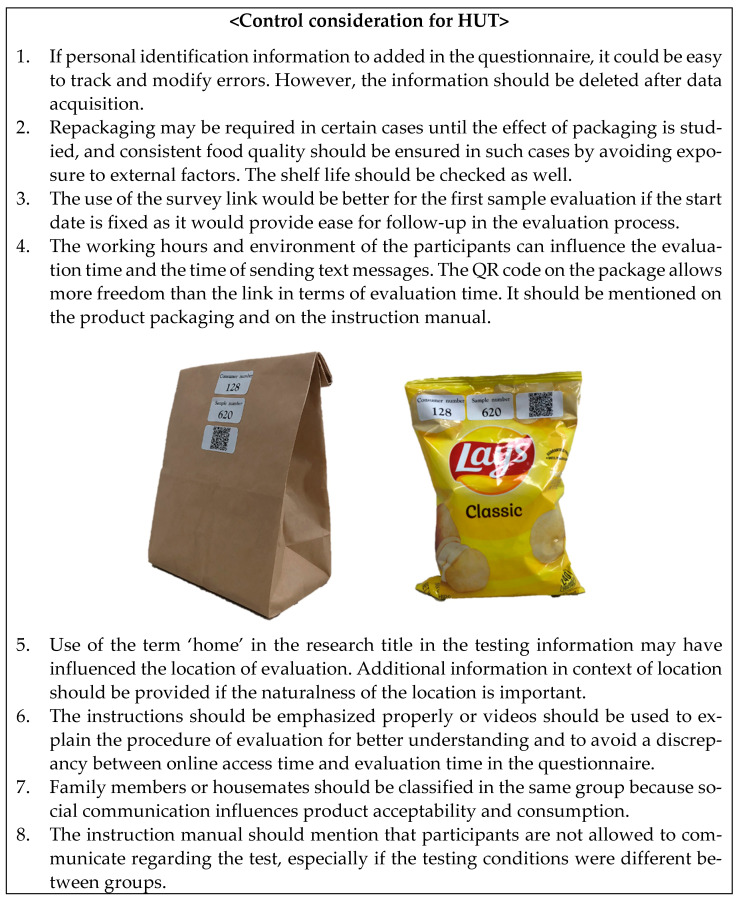
A summary of instructions in HUT.

Social communication was not considered in our study. Snacks are generally eaten alone, however, their acceptability and consumption can be influenced by social interaction. In our study, family members or housemates were classified in the same group. However, this factor was excluded from the analysis due to low occurrences. Furthermore, some participants who were acquaintances contacted each other regarding the testing, although they were not in the same group during the evaluation period. Thus, better instruction was needed to avoid communication among participants for reduction of errors, such as participants from the ‘Time and order control’ group receiving survey links from the ‘Order control’ group. The recommendations for HUT is summarized in the Figure 5.

## 4. Conclusions

This study investigated and compared the results of three groups differing in time and order control using six samples with different textures in the home use test. It aimed to determine the amount of control/effort that would be required to handle errors that might occur while conducting HUT. Overall, the results of the evaluation were similar between the groups, regardless of the degree of control. Thus, HUT can be utilized similarly to CLT as consumer tests in terms of the number of samples. HUT allows the evaluation of samples in a real environment and can be designed to evaluate long-term usage. They can be utilized to improve the launch of new products and evaluate their success.

Not much research has been conducted in a realistic environment and there is almost no report on errors that may occur while conducting HUT. However, a few of its disadvantages are its cost and high dropout rate. This study evaluated two control factors of preassigned order and evaluation time in HUT. It included the evaluation of six snack samples by participants, which is normally evaluated in one session in CLT or laboratory evaluation. If CLT or laboratory tests were included as a control compared to HUT, our findings would have been better validated. As a small number of consumer sample participated in this study, our findings may not be generalized. When conducting HUT with more consumers providing a higher number of samples than traditional HUT of one or two samples, more errors or higher dropout rate might be observed. More experiments on HUT should be performed for generalization. Furthermore, other control factors, such as sample temperature, should also be considered in the future.

## Figures and Tables

**Table 1:** Demographic information of the three groups of consumers.

Variables	No Control		Order Control		Time and Order Control		Total	
	*N*	%	*N*	%	*N*	%	*N*	%
**Sex**								
Female	70(3) ^1^	70.0	74(2)	74.0	74	74.0	218(5)	72.7
Male	30(2)	30.0	26	26.0	26(1)	26.0	82(3)	27.3
**Age**								
19–25	32(3)	32.0	28	28.0	30	30.0	90(3)	30.0
26–35	51(2)	51.0	51(1)	51.0	51(1)	51.0	153(4)	51.0
36–45	13	13.0	13(1)	15.0	12	12.0	38(1)	12.7
46–55	3	3.0	5.0	5.0	5	5.0	13	4.3
56–65	1	1.0	3.0	3.0	2	2.0	6	2.0
**Job** ^2^								
Student	33	34.7	25	25.5	25	25.3	83	28.4
Office worker	47	49.5	52	53.1	55	55.6	154	52.7
Self-employed	1	1.1	4	4.1	1	1.0	6	2.1
Housewife	3	3.2	6	6.1	7	7.1	16	5.5
Not working	7	7.4	8	8.2	9	9.1	24	8.2
Others	4	4.2	3	3.1	2	2.0	9	3.1
**Snacking frequency per day**								
0	2	2.1	3	3.1	0	0.0	5	1.7
1	59	62.1	54	55.1	61	61.6	174	59.6
2	27	28.4	30	30.6	30	30.3	87	29.8
3	5	5.3	7	7.1	4	4.0	16	5.5
≥4	2	2.1	4	4.1	4	4.0	10	3.4
**Usual snacking time**								
Between breakfast and lunch	23	16.1	28	20.0	18	12.9	69	16.3
Between lunch And dinner	78	54.5	74	52.9	71	50.7	223	52.7
Between post dinner and before sleeping	42	29.4	38	27.1	51	36.4	131	31.0

^1^ The number in parentheses indicates consumers who dropped out of study. ^2^ Job and snacking question for the participants of ‘No control’ (*n* = 95), ‘Order control’ (*n* = 98), and ‘Time and order control’ (*n* = 99) groups were asked at the end of evaluation, thus the number between the groups differed.

**Table 2:** Information of six samples evaluated.

Label	Product	Manufacturer	Amount Per Package	Recommended Serving Size on Package	Units Provided	Weight Provided	Price for Quantity Provided (USD) ^1^	Mouth Behavior
Apple sauce	Mott’s^®^ Applesauce Apple	Mott’s, LLP, Plano, TX, USA	113 g	113 g	1 container	113 g	0.59	Smoosher
Candy	Ricola Lemon Mint (sugar free)	Ricola Ltd., Laufen, Switzerland	342 g	3.6 g	4 drops	14.4 g	0.35	Sucker
Cereal bar	Kellogg’s^®^ Rice Krispies Treats^®^	Kellogg, Battle Creek, MI, USA	22 g	22 g	2 bars	44 g	0.59	Chewer
Jelly	HARIBO Mega-Roulette	Haribo, Solingen, Germany	45 g	45 g	1 package	45 g	0.38	Chewer
Potato chip	LAY’S^®^ Classic Potato Chips	Frito-Lay, INC., Plano, TX, USA	42.5 g	42.5 g	1 package	42.5 g	1.09	Cruncher
Spread wafer	Nutella B-ready	Ferrero OHG mbH, Hessen, Germany	22 g	22 g	2 bars	44 g	1.47	Cruncher & Smoosher

Symbol: ^®^—Stands for Registered Trademark. ^1^ Exchange rate at 1230 KRW for 1 USD (as of May 2020).

**Table 3:** Frequency of available data used, complete data and error data that were modifiable in each group ^1^.

Group	No Control	Order Control	Time and Order Control
**Completed without error**			
Apple sauce	92	69	80
Candy	89	79	77
Cereal bar	85	79	81
Jelly	88	77	83
Potato chip	97	73	83
Spread wafer	90	80	95
**Error occurrence frequency** **(Time, Evaluation Order, Sample number error)**			
Apple sauce	8	36	20
Candy	11	28	23
Cereal bar	15	22	20
Jelly	12	23	17
Potato chip	3	30	18
Spread wafer	10	21	7
**Recovered error**			
Apple sauce	3	28	19
Candy	7	19	22
Cereal bar	12	18	18
Jelly	8	22	16
Potato chip	3	24	17
Spread wafer	9	18	4
**No response**			
Apple sauce	5	3	1
Candy	4	2	1
Cereal bar	3	3	1
Jelly	4	1	1
Potato chip	0	3	0
Spread wafer	1	2	1
**Total number of dropped consumers**	5	2	1
**Extended evaluation**	7	8	57
**Final number of consumers for data analysis ^2^**			
Apple sauce	95	97	99
Candy	96	98	99
Cereal bar	97	97	99
Jelly	96	99	99
Potato chip	100	97	100
Spread wafer	99	98	99

^1^ Frequency indicates number of consumers having one or more error in each sample evaluation. ^2^ Final completed number of consumers are 100 minus ‘no response’. All other errors were recoverable with confirmation.

**Table 4:** Information of evaluation time and place for each consumer group ^1^.

Group	Time								Place of Consumption							
	Morning (6 a.m.–12 p.m.)		Afternoon (12 p.m.–6 p.m.)		Evening (6 p.m.–12 a.m.)		Dawn (12 a.m.–6 a.m.)		Home		Work Place		School		Others	
	*N*	%	*N*	%	*N*	%	*N*	%	*N*	%	*N*	%	*N*	%	*N*	%
**No control**																
Apple sauce	13	13.7	31	32.6	48	50.5	3	3.2	79	83.2	13	13.7	2	2.1	1	1.1
Candy	20	21.1	33	34.7	36	37.9	7	7.4	69	71.9	17	17.7	4	4.2	6	6.3
Cereal bar	16	16.8	38	40.0	40	42.1	3	3.2	83	85.6	10	10.3	3	3.1	1	1.0
Jelly	14	14.7	41	43.2	34	35.8	7	7.4	79	82.3	10	10.4	0	0.0	7	7.3
Potato chip	16	16.8	30	31.6	47	49.5	7	7.4	88	88.0	8	8.0	2	2.0	2	2.0
Spread wafer	24	25.3	32	33.7	38	40.0	5	5.3	76	76.8	20	20.2	2	2.0	1	1.0
**Order control**																
Apple sauce	31	32.0	25	25.8	34	35.1	7	7.2	80	81.6	14	14.3	3	3.1	1	1.0
Candy	26	26.8	32	33.0	35	36.1	5	5.2	76	76.8	15	15.2	3	3.0	5	5.1
Cereal bar	25	25.8	26	26.8	43	44.3	3	3.1	76	77.6	17	17.3	2	2.0	3	3.1
Jelly	22	22.7	36	37.1	37	38.1	4	4.1	75	75.8	15	15.2	4	4.0	5	5.1
Potato chip	13	13.4	29	29.9	46	47.4	9	9.3	83	85.6	7	7.2	3	3.1	4	4.1
Spread wafer	25	25.8	26	26.8	41	42.3	6	6.2	83	84.7	10	10.2	3	3.1	2	2.0
**Time and order control**																
Apple sauce	29	29.3	30	30.3	37	37.4	3	3.0	79	79.8	17	17.2	1	1.0	2	2.0
Candy	30	30.3	34	34.3	32	32.3	3	3.0	76	76.8	15	15.2	3	3.0	5	5.1
Cereal bar	34	34.3	28	28.3	35	35.4	2	2.0	75	75.8	18	18.2	2	2.0	4	4.0
Jelly	33	33.3	30	30.3	34	34.3	2	2.0	77	77.8	16	16.2	3	3.0	3	3.0
Potato chip	26	26.3	30	30.3	42	42.4	2	2.0	83	83.0	13	13.0	2	2.0	2	2.0
Spread wafer	29	29.3	28	28.3	36	36.4	6	6.1	81	81.8	15	15.2	0	0.0	3	3.0

^1^ The number of participants who completed the test differs depending on the group. The number of participants for each group were as follows: ‘No control’ (*n* = 95), ‘Order control’ (*n* = 98), and ‘Time and order control’ (*n* = 99). Thus, the percentage was added for comparison.

**Table 5:** Number of days ^1^ taken for the home use test (HUT) with six samples.

Group	No Control	Order Control	Time and Order Control
Mean (±SD)	9.0 ± 4.1	9.0 ± 3.8	15.0 ± 2.7
Minimum	1.0	1.0	10.0
Median	9.0	9.0	15.0
Maximum	26.0	18.0	25.0

Abbreviation: SD—standard deviation. ^1^ Value of the difference between start and end date using date unit (‘Time and order control’ had fixed interval evaluation).

**Table 6:** Consumer’s liking and perceived intensity of ‘No control’, ‘Order control’, and ‘Time and order control’ groups ^1, 2, 3^.

Sample	Overall	Package	Flavor	Texture	Afterfeel	Afterfeel Intensity	Texture Intensity	Amount of Residue
**No control**								
Apple sauce	4.8 ^d^	5.5 ^c^	5.7 ^d^	4.8 ^c^	4.9 ^c^	5.6 ^b^	2.6 ^f^	1.6 ^c^
Candy	6.3 ^c^	6.2 ^ab^	6.6 ^b^	6.3 ^b^	6.3 ^a^	6.3 ^a^	7.4 ^a^	1.1 ^d^
Cereal bar	6.2 ^c^	6.0 ^b^	6.1 ^cd^	6.5 ^b^	5.6 ^b^	6.1 ^a^	4.2 ^e^	2.2 ^b^
Jelly	6.5 ^bc^	6.0 ^b^	6.4 ^bc^	6.1 ^b^	5.8 ^b^	5.6 ^b^	6.5 ^b^	1.3 ^cd^
Potato chip	6.9 ^ab^	6.1 ^b^	6.6 ^b^	7.4 ^a^	5.5 ^b^	6.2 ^a^	5.2 ^c^	2.5 ^ab^
Spread wafer	7.2 ^a^	6.6 ^a^	7.1 ^a^	6.5 ^b^	5.5 ^b^	6.2 ^a^	4.6 ^d^	2.7 ^a^
*p* value	<0.0001	<0.0001	<0.0001	<0.0001	<0.0001	<0.0001	<0.0001	<0.0001
LSD	0.45	0.37	0.42	0.43	0.45	0.37	0.40	0.29
**Order control**								
Apple sauce	4.5 ^c^	5.4 ^b^	5.3 ^e^	4.5 ^d^	4.5 ^c^	6.0 ^bc^	2.2 ^f^	1.6 ^c^
Candy	6.3 ^b^	6.4 ^a^	6.5 ^bc^	6.1 ^c^	6.2 ^a^	6.4 ^a^	7.3 ^a^	0.9 ^e^
Cereal bar	6.2 ^b^	5.7 ^b^	6.1 ^d^	6.3 ^c^	5.4 ^b^	6.1 ^bc^	3.9 ^e^	2.2 ^b^
Jelly	6.3 ^b^	6.2 ^a^	6.5 ^cd^	6.0 ^c^	5.6 ^b^	5.8 ^c^	6.6 ^b^	1.3 ^d^
Potato chip	7.1 ^a^	6.2 ^a^	6.9 ^ab^	7.4 ^a^	5.7 ^b^	6.1 ^abc^	5.1 ^c^	2.5 ^a^
Spread wafer	7.3 ^a^	6.6 ^a^	7.0 ^a^	6.8 ^b^	5.5 ^b^	6.3 ^ab^	4.4 ^d^	2.7 ^a^
*p* value	<0.0001	<0.0001	<0.0001	<0.0001	<0.0001	<0.0001	<0.0001	<0.0001
LSD	0.48	0.39	0.44	0.48	0.47	0.36	0.41	0.28
**Time and order control**								
Apple sauce	5.0 ^c^	5.6 ^c^	5.5 ^c^	4.7 ^d^	5.0 ^c^	5.7 ^c^	2.1 ^f^	1.5 ^c^
Candy	6.2 ^b^	6.1 ^ab^	6.4 ^b^	6.0 ^c^	6.1 ^a^	6.3 ^a^	7.4 ^a^	1.2 ^d^
Cereal bar	6.4 ^b^	5.8 ^bc^	6.3 ^b^	6.5 ^b^	5.4 ^bc^	6.1 ^ab^	3.9 ^e^	2.3 ^b^
Jelly	6.3 ^b^	5.7 ^c^	6.3 ^b^	5.6 ^c^	5.5 ^b^	5.7 ^c^	6.5 ^b^	1.3 ^cd^
Potato chip	6.9 ^a^	6.2 ^a^	6.8 ^a^	7.2 ^a^	5.4 ^bc^	5.9 ^bc^	5.3 ^c^	2.5 ^ab^
Spread wafer	7.1 ^a^	6.4 ^a^	7.1 ^a^	6.7 ^b^	5.4 ^bc^	6.0 ^abc^	4.7 ^d^	2.6 ^a^
*p*-value	<0.0001	<0.0001	<0.0001	<0.0001	<0.0001	<0.0001	<0.0001	<0.0001
LSD	0.45	0.37	0.39	0.49	0.45	0.36	0.39	0.29

Abbreviation: LSD—least significant difference. ^1^ Evaluated using the nine-point scale from 1 (dislike extremely) to 9 (like extremely) and the amount of residue was rated using the six-point scale from 0 (none) to 5 (very much). ^2^ A lower case alphabet indicates significant differences within each group (α = 0.05). ^3^ There was no significant difference between groups (α = 0.05).

**Table 7:** Knowledge of samples ^1^.

Sample	Product Awareness		Brand Awareness		Experience		Total
	*N*	%	*N*	%	*N*	%	*N*
**No control**							
Apple sauce	4	4.2	4	4.2	3	3.2	95
Candy	53	55.2	42	43.8	49	51.0	96
Cereal bar	44	45.4	55	56.7	41	42.3	97
Jelly	68	70.8	88	91.7	58	60.4	96
Potato chip	74	74.0	61	61.0	57	57.0	100
Spread wafer	34	34.3	88	88.9	20	20.2	99
**Order control**							
Apple sauce	7	7.2	6	6.2	5	5.2	97
Candy	57	58.2	47	48.0	53	54.1	98
Cereal bar	45	46.4	62	63.9	39	40.2	97
Jelly	79	79.8	95	96.0	61	61.6	99
Potato chip	71	73.2	56	57.7	57	58.8	97
Spread wafer	31	31.6	84	85.7	22	22.4	98
**Time and order control**							
Apple sauce	8	8.1	7	7.1	6	6.1	99
Candy	51	51.5	34	34.3	46	46.5	99
Cereal bar	44	44.4	67	67.7	41	41.4	99
Jelly	78	78.8	88	88.9	63	63.6	99
Potato chip	73	73.0	67	67.0	60	60.0	100
Spread wafer	26	26.3	86	86.9	18	18.2	99

^1^ The frequency of awareness, brand awareness, and experience were measured using Yes or No.

**Table 8:** Evaluated order frequency for each sample in the ‘No control’ group.

Sample	1	2	3	4	5	6	Cumulative Evaluation (*N*)
Apple sauce	6	7	6	11	23	42	95
Candy	7	6	9	28	21	25	96
Cereal bar	11	17	28	18	15	8	97
Jelly	14	20	22	15	17	8	96
Potato chip	47	19	13	8	7	6	100
Spread wafer	15	29	19	17	13	6	99
Total (N) ^1^	100	98	97	97	96	95	

^1^ The frequency of the chosen sample each time from 1 to 6.

**Table 9:** Purchase intent and price willing to pay.

Sample	Purchase Intent ^1^			Appropriate Price (USD) ^2^					
	No Control	Order Control	Time and Order Control	No Control		Order Control		Time and Order Control	
				Mean	SD	Mean	SD	Mean	SD
Apple sauce	2.3 ^c,3^	1.9 ^d^	2.1 ^d^	1.02 ^ab^	0.48	1.09 ^ab^	0.53	1.08 ^a^	0.55
Candy	3.3 ^b^	3.4 ^ab^	3.0 ^c^	0.41 ^e^	0.48	0.44 ^e^	0.48	0.50 ^d^	0.54
Cereal bar	3.2 ^b^	3.0 ^c^	3.1 ^bc^	0.80 ^d^	0.34	0.75 ^d^	0.39	0.73 ^c^	0.36
Jelly	3.3 ^ab^	3.2 ^bc^	3.2 ^bc^	1.06 ^a^	0.40	1.15 ^a^	0.55	1.11 ^a^	0.46
Potato chip	3.3 ^b^	3.5 ^ab^	3.4 ^ab^	0.91 ^c^	0.29	0.99 ^bc^	0.33	1.00 ^a^	0.35
Spread wafer	3.6 ^a^	3.5 ^a^	3.6 ^a^	0.93 ^bc^	0.36	0.96 ^c^	0.42	0.88 ^b^	0.40
*p*-value	<0.0001	<0.0001	<0.0001	<0.0001		<0.0001		<0.0001	
LSD	0.32	0.31	0.31	0.10		0.11		0.11	

Abbreviation: LSD—least significant difference; SD—standard deviation. ^1^ Purchase intent was measured using the five-point scale from 1 = definitely would not purchase to 5 = definitely would purchase. ^2^ Appropriate price was asked as an open-ended question in KRW. And exchange rate of 1230 KRW was used to calculate the price in USD (as of May 2020). Converted price was used for the analysis. ^3^ Sharing the same lower case letter means there is no significant difference between samples (α = 0.05). There was no significant difference between groups (α = 0.05).

**Table 10:** Mouth behavior of the consumers used during consumption.

Sample	Cruncher		Chewer		Sucker		Smoosher		Total
	*N*	%	*N*	%	*N*	%	*N*	%	*N*
**Total**									
Apple sauce	7	2.4	114	38.4	130	43.8	46	15.5	297
Candy	100	19.3	91	17.6	272	52.5	55	10.6	518
Cereal bar	95	20.0	278	58.6	13	2.7	88	18.6	474
Jelly	11	2.5	285	64.5	81	18.3	65	14.7	442
Potato chip	186	35.1	272	51.3	10	1.9	62	11.7	530
Spread wafer	177	33.0	258	48.1	36	6.7	65	12.1	536
**No control**									
Apple sauce	0	0.0	31	32.0	53	54.6	13	13.4	95
Candy	31	19.5	20	12.6	89	56.0	19	11.9	96
Cereal bar	26	17.1	92	60.5	5	3.3	29	19.1	97
Jelly	3	2.0	93	63.3	30	20.4	21	14.3	96
Potato chip	70	38.0	89	48.4	1	0.5	24	13.0	100
Spread wafer	55	32.9	82	49.1	10	6.0	20	12.0	99
**Order control**									
Apple sauce	2	2.2	40	44.0	34	37.4	15	16.5	97
Candy	36	19.7	34	18.6	94	51.4	19	10.4	98
Cereal bar	30	19.4	93	60.0	4	2.6	28	18.1	97
Jelly	5	3.4	96	65.8	24	16.4	21	14.4	99
Potato chip	58	35.2	90	54.5	3	1.8	14	8.5	97
Spread wafer	59	32.6	88	48.6	11	6.1	23	12.7	98
**Time and order control**									
Apple sauce	5	4.6	43	39.4	43	39.4	18	16.5	99
Candy	33	18.8	37	21.0	89	50.6	17	9.7	99
Cereal bar	39	23.4	93	55.7	4	2.4	31	18.6	99
Jelly	3	2.0	96	64.4	27	18.1	23	15.4	99
Potato chip	58	32.0	93	51.4	6	3.3	24	13.3	100
Spread wafer	63	33.5	88	46.8	15	8.0	22	11.7	99

**Table 11:** The amount of consumption evaluated by consumers.

Group	Less Than 1/3		1/3		1/2		3/4		All		Total
	*N*	%	*N*	%	*N*	%	*N*	%	*N*	%	*N*
**No control**											
Apple sauce	22	23.2	23	24.2	8	8.4	5	5.3	37	38.9	95
Candy	20	20.8	18	18.8	30	31.3	1	1.0	27	28.1	96
Cereal bar	2	2.1	9	9.3	29	29.9	5	5.2	52	53.6	97
Jelly	7	7.3	36	37.5	14	14.6	6	6.3	33	34.4	96
Potato chip	4	4.0	16	16.0	12	12.0	8	8.0	60	60.0	100
Spread wafer	4	4.0	6	6.1	34	34.3	2	2.0	53	53.5	99
**Order control**											
Apple sauce	21	21.6	24	24.7	14	14.4	2	2.1	36	37.1	97
Candy	27	27.6	22	22.4	24	24.5	0	0.0	25	25.5	98
Cereal bar	4	4.1	2	2.1	37	38.1	3	3.1	51	52.6	97
Jelly	14	14.1	35	35.4	18	18.2	7	7.1	25	25.3	99
Potato chip	4	4.1	18	18.6	11	11.3	8	8.2	56	57.7	97
Spread wafer	3	3.1	7	7.1	29	29.6	3	3.1	56	57.1	98
**Time and order control**											
Apple sauce	12	12.1	29	29.3	15	15.2	7	7.1	36	36.4	99
Candy	30	30.3	16	16.2	27	27.3	4	4.0	22	22.2	99
Cereal bar	5	5.1	5	5.1	35	35.4	2	2.0	52	52.5	99
Jelly	13	13.1	37	37.4	15	15.2	5	5.1	29	29.3	99
Potato chip	1	1.0	24	24.0	7	7.0	12	12.0	56	56.0	100
Spread wafer	3	3.0	6	6.1	28	28.3	1	1.0	61	61.6	99
